# Influence of bisphosphonate therapy on bone geometry, volumetric bone density and bone strength of femoral shaft in postmenopausal women with rheumatoid arthritis

**DOI:** 10.1186/s12891-016-1167-8

**Published:** 2016-08-05

**Authors:** Rahel Meinen, Inna Galli-Lysak, Peter M. Villiger, Daniel Aeberli

**Affiliations:** Department of Rheumatology, Immunology and Allergology, Inselspital Bern, University Hospital Bern, Bern, Switzerland

**Keywords:** Femoral bone, pQCT, Bisphosphonates, RA, Bone strength, AFF

## Abstract

**Background:**

There is evidence that postmenopausal women with rheumatoid arthritis (RA) on glucocorticoid (GC) therapy and bisphosphonate (BP) have an increased risk for atypical subtrochanteric and atypical diaphyseal femoral fracture (AFF). The underlying mechanism has not been elucidated so far. Using peripheral quantitative computed tomography (pQCT), the aim of the present study was to compare bone geometry, volumetric bone mineral density (vBMD) and bone strength of femoral shaft in BP-treated and BP-naïve postmenopausal women with RA.

**Methods:**

Prospective cross-sectional pQCT scans were taken at 33 % of total femur of BP-treated and BP-naïve RA patients. Bone parameters of the two groups were compared and correlated to disease characteristics and muscle cross-sectional area (CSA).

**Results:**

A total of 60 consecutive postmenopausal RA patients, 20 with BP therapy and 40 BP-naïve, were included in the study. The median age of the subjects was 63.5 years (range 48–85 years), and median disease duration (RA) was 12.0 years (range 2–47 years). Height and weight of the patients of the two groups were comparable. Women in the BP group were on average 4.3 years older (*p* = 0.044), and duration since menopause was on average 5.76 years longer (*p* = 0.045). In the BP group, there was a 13.31 % reduced muscle cross-sectional area around the proximal thigh (*p* = 0.013); cortical CSA was smaller by 5.3 % (*p* = 0.043); however, total and medullary CSA, as well as cortical vBMD and the polar bone stress–strain index of the femoral shaft were similar in the two groups. In regression analysis, age, time since menopause and muscular CSA were significant factors determining cortical CSA, cortical thickness and femoral index (*p* < 0.05). Regression model showed no significant effect of BP therapy on bone geometry and density of the femoral diaphysis at 33 %.

**Conclusion:**

Differences in cortical CSA between BP-treated and BP-naïve postmenopausal RA patients were found to be associated only with differences in age, time since menopause and muscle cross-sectional area around the proximal thigh. In interpreting our results, it should be kept in mind that BP was given only to patients with increased fracture risk. This fact might have a confounding effect on our findings of differences between the two groups.

## Background

In rheumatoid arthritis (RA), bone damage such as erosions, juxta-articular osteopenia, as well as generalized osteopenia and osteoporosis are well recognized [1]. Decreased cortical thickness and enlarged total cross-sectional area have recently been described in the shaft of the metacarpal bone, radius and tibia [2]. In order to prevent further bone loss, antiresorptive therapies are applied to patients with diagnosed osteoporosis or with increased fracture risk. Whereas the effect of anti-resorptives, particularly bisphosphonate (BP) on trabecular bone such as spine and femoral head is clearly defined and its use leads to a prevention of peripheral and vertebral fractures, the effect on bone shaft geometry is subject to controversy. Potential complications of long-term BP use, such as atypical femoral fracture (AFF) of the diaphysis raise questions about the suitability of its use over time, particularly in patients with active RA with concomitant use of glucocorticoid (GC), and low vitamin D levels [3]. Clinical data support a BP-induced increased risk of AFF, as shown by a 70 % reduction in AFF one year after BP withdrawal [4].

Peripheral quantitative computed tomography (pQCT) is a three-dimensional measuring technique that allows the assessment of cross-sectional bone geometry and volumetric bone mineral density (vBMD). Because bone shaft geometry mainly results from mechanical force, aging, duration and therapy of RA [5–7] and therapy-induced alterations in bone shaft geometry are challenging to measure [8], pQCT is more advantageous compared to 2-dimensional methods such as dual x-ray absorptiometry (DXA) that permit measurement of bone geometry only in the projected plane and is dependent on bone rotation, tissue and calcification [9]. So far, when pQCT was used in investigations of RA patients, the bones assessed were the tibia, the radius and the metacarpal bone [2, 10–14]. To date, no study has examined true volumetric BMD and bone geometry of femoral bones in RA.

The aim of the present study was, therefore, to compare bone properties measured at the femoral diaphysis of RA patients treated with bisphosphonate with those of RA patients who did not undergo such therapy.

## Methods

We conducted a prospective cross-sectional study to evaluate the effect of BP therapy on femoral bone geometry, volumetric density and bone strength in a cohort of postmenopausal RA patients treated with BP compared to a BP-naïve control RA cohort. BP therapy was given to patients with osteoporosis, increased fracture-risk or as prevention of glucocorticoid-induced osteoporosis in case of long-term high dose glucocorticoid use.

From November 2013 to September 2014, consecutive postmenopausal women with RA and > 45 years of age were recruited for the study at the Department of Rheumatology, University of Bern, Switzerland. They fulfilled the American College of Rheumatology criteria for RA [15]. Data on disease duration, modified disease activity score (DAS) including 28 joints [16], and therapy with bisphosphonate and glucocorticoids were extracted. Decision on use of BP had been made earlier by the treating rheumatologist, general practitioner or expert in osteoporosis according to the guidelines of the IOF, ACR and ASBMR [17–19].

Exclusion criteria were as follows: hyperparathyroidism, substance abuse (alcohol), hepatitis, HIV, malignancies, neuropathies, previous or present use of parathyroid hormone, anabolic steroid or growth hormone within 6 months before trial entry, currently under hormone replacement therapy, anabolic osteoporosis treatment, and creatinine clearance <30 ml/min at baseline.

vBMD and geometry of the femur as well as thigh muscle CSA were measured using pQCT. Measurements of the femur are described in detail elsewhere [20, 21]. In short, pQCT measurements were performed at the non-dominant leg (usually on the contralateral side of the non-dominant hand) using the Stratec XCT300 scanner (Stratec, Germany). A scout view was performed at the distal femur end. On the scout view, a reference line was placed at the distal end of the respective bone. Scans of the femoral diaphysis were taken at 33 % proximal to the reference line. Each scan was acquired at a slice thickness of 2 mm. Voxel size was 0.3 mm edge length with a scanning speed of 15 mm/s at the femur. Image processing and calculation of the various bone parameters were performed automatically using the manufacturer’s software package (version 5.5D). Cortical bone properties were assessed at the diaphyseal sites (33 %) using the default threshold value of 710 mg/cm^3^. The following parameters were assessed at the diaphyseal site: total bone CSA (including medullary CSA), cortical CSA (excluding medullary CSA), medullary CSA (total CSA minus cortical CSA), and cortical volumetric BMD (mg/cm3). Assuming the bone shaft to be cylindrical, cortical thickness was calculated from total CSA, which included the bone marrow, and cortical CSA of the diaphyseal scans. The polar bone stress–strain index (SSI; in mm3) is a measure of diaphyseal bone resistance to bending and torsion [22–24] and can be used as a surrogate for bone strength [24, 25]. It was derived from the diaphyseal scans, which were also used to quantify muscle CSA of the thigh by measuring total leg CSA and subtracting CSAs of bone and fat. Analogous to the metacarpal index, we calculated a femoral index [26].

IBM SPSS Statistics 21 was used for statistical evaluations. The significance level of the tests was set at 5 %. Patient characteristics and outcomes were compared between the groups with non-parametric Mann–Whitney U tests. Multivariable linear regression was used to adjust the analysis for cortical thickness, cortical CSA, femoral index, and polar SSI for possible confounders such as age, time since menopause, disease duration, and muscle area. Normality of the residuals was checked by p-p-plots.

## Results

Between 2011 and 2014, 94 consecutive RA patients were invited to participate in the study; a total of 34 were excluded from the study as follows: premenopausal state at age > 45 (*n* = 13), malignoma (*n* = 5), ongoing hormone replacement therapy (*n* = 6), inadequate RA diagnosis (*n* = 2), insufficient scan quality (*n* = 2), thyroid disorders (*n* = 3), hepatitis (*n* = 1), neuropathy (*n* = 1) or osteoanabolic therapy with PTH (*n* = 1).

Thus, 60 patients were included in our analysis; 20 patients were under treatment with BP, while 40 patients were BP-naïve. Seven patients had euthyroid hypothyroidism with substitution therapy. Patient characteristics of the two groups are shown in Table 1.

**Table 1:** Patient characteristics

	BP-naïve group				BP group				
	N	Median	Mean	95 %-CI of mean	N	Median	Mean	95 %-CI of mean	*p*-value
Age (years)	40	59.5	61.9	59.14-64.66	20	67.5	66.2	62.2-70.3	0.044
Height (cm)	39	163.0	164.0	161.7-166.3	20	160.5	160.7	157.3-164.1	0.159
Weight (kg)	38	70.0	71.61	67.2-76.0	20	69.5	68.6	63.3-74.2	0.584
Muscle area at 33 % of femur (mm^2^)	40	6392.4	6381.7	6055.5-6708.0	20	5291.2	5632.0	5094.2-6169.9	0.013
Age at menopause (yrs)	38	50.00	48.47	46.28-50.67	19	48.0	47.3	44.32-50.31	0.329
Time since menopause (yrs)	38	14.00	15.03	11.99-18.07	19	21.0	20.8	15.65-25.93	0.045
Duration of RA (yrs)	39	11.00	13.67	10.74-16.59	17	15.0	21.1	14.32-27.92	0.040

To compare disease activity between the groups, DAS28 score was analyzed if available in the patients’ records. DAS28 score overall was low (median 2.9) and was 7.0 % lower in the BP group (*p* = 0.537). Ninety percent in the BP group were on disease-modifying antirheumatic drug (DMARD) with 40 % being on methotrexate (MTX), and 55 % on a biological. In the BP-naïve group, 85 % were on a DMARD with 50 % being on MTX, while 37.5 % were treated with a biological. In the BP group, the median duration of BP therapy was 1095 days (mean: 1171 days, 95 % CI 843–1499); in half of the patients bisphosphonates were orally administered. At the time when bone properties were measured, in the BP group, 50 % were receiving GC therapy with an average dose of 2.28 mg/d; in the BP-naïve group, 47 % were receiving GC therapy with an average dose of 2.00 mg/d (*p* > 0.05). However, data on long-term GC use and dosing were too meager to be used for further analysis. 25-OH-vitamin D3 levels were between 50-90 nmol/l, in the BP group with 85 % of the patients receiving vitamin D supplementation compared to 62.5 % in the BP-naïve group. Total calcium intake was approximately 1000 mg/d in both groups; calcium was supplemented in 60 % of the patients of the BP group as against 55 % in BP-naïve group.

In the crude analysis, median cortical CSA was 5.3 % lower (*p* = 0.043) in the BP group. Similar pattern was found for median cortical thickness and the polar bone stress–strain index (4.8 %, *p* = 0.076 and 13.7 %, *p* = 0.082 respectively; Table 2).

**Table 2:** Outcomes, depicted as median, mean, and 95 % CI of the mean. (not adjusted for age, muscle area, time since menopause, and duration of RA)

	BP-naïve group				BP group				
	N	Median	Mean	95 %-CI of mean	N	Median	Mean	95 %-CI of mean	*p*-value
Cortical CSA (mm^2^) at 33 % of femur	40	314.43	323.22	308.72-337.73	20	298.61	296.43	284.70-308.17	0.043
Cortical thickness in mm at 33 % of the femur	40	4.209	4.285	4.054-4.517	20	4.017	3.886	3.597-4.174	0.076
Medullary CSA (mm^2^) at 33 % of femur	40	180.40	191.66	172.82-210.49	20	196.68	227.65	177.84-277.45	0.466
Medullary CSA in relation to total CSA (%)	40	29.47	29.62	27.48-31.77	20	31.06	33.71	29.49-37.92	0.113
Cortical vBMD (mg/cm^3^) at 33 % of femur	40	1061.86	1062.03	1046.9-1077.1	20	1067.86	1036.03	994.02-1078.05	0.562
Total bone CSA (mm^2^) at 33 % of femur	40	623.11	639.91	613.87-665.94	20	643.31	649.58	586.36-712.81	0.925
Femoral index (%)	40	29.791	30.279	28.406-32.12	20	28.897	27.769	24.672-30.865	0.227
Polar SSI (mm3)	40	2640.840	2590.723	2411.798- 2769.648	20	2321.920	2277.980	2008.614- 2547.347	0.082

In order to compare the individual ratio of the CSA of femoral bone to cortical thickness, we calculated the relation of medullary CSA to total femoral bone CSA as well as the femoral index. We found no difference between the relation of medullary CSA to total femoral bone CSA and femoral index between the two groups (5.1 %, *p* = 0.113 and 3.1 %, *p* = 0.227 respectively; Table 2). In order to control for the influence of other factors, linear regression models were fitted including potential confounders identified in the crude group comparison (age, time since menopause, duration of RA and muscle CSA). Cortical CSA, cortical thickness, femoral index and polar SSI were set as outcomes (Table 3).

**Table 3:** Summary of linear regression models for cortical thickness, cortical CSA, and femoral index

	Cortical CSA		Cortical thickness		Femoral index		Pol SSI	
	β	*p*-value	β	*p*-value	β	*p*-value	β	*p*-value
Age	0.570	0.004	0.596	0.003	0.505	0.015	−0.066	0.763
Time since menopause	−0.414	0.032	−0.541	0.006	−0.525	0.010	0.121	0.577
Duration of RA	0.029	0.828	−0.079	0.550	−0.122	0.384	−0.090	0.558
Muscle CSA	0.398	0.004	0.375	0.006	0.305	0.032	0.122	0.424
BP	−0.251	0.071	−0.189	0.170	−0.115	0.425	−0.235	0.140

No multivariate regression model showed a significant influence of BP therapy on bone geometry and density of the femoral diaphysis at 33 %. Polar SSI at 33 % of femoral diaphysis was not associated with any of the explanatory variables (Table 3).

## Discussion

In this prospective cross-sectional study, we evaluated bone geometry, density and bone strength of the femoral diaphysis at 33 % in 20 postmenopausal RA patients treated with BP, comparing them with a control group of 40 postmenopausal BP-naïve RA patients. Measurements were performed using pQCT. We found the cortical CSA of femoral bone to be lower in BP-treated than in BP-naïve RA patients (*p* = 0.043). Cortical thickness tended to be lower (*p* = 0.076), medullary cavity, total femoral shaft CSA, cortical vBMD, and polar bone SSI were not significantly different between the groups. In a regression analysis, muscle cross-sectional area around the proximal thigh, age and time since menopause were factors significantly determining cortical thickness, cortical CSA and femoral index. No regression model showed a significant influence of BP therapy on bone geometry and density of the femoral diaphysis at 33 %.

To our knowledge, this is the first prospective cross-sectional study comparing femoral bone geometry in postmenopausal RA patients treated with BP and BP-naïve postmenopausal RA patients. So far, only a few studies have investigated bone shaft geometry in RA patients [2, 6, 12, 27–29]. Recently, in a prospective study, we evaluated bone geometry and density at the third metacarpal bone, the tibia and the radius by means of pQCT scans. We found RA patients to have significantly thinner cortices, whereas total CSA of the outer metacarpal bone shaft was increased as compared to healthy controls [2]. In that study, we found age-associated cortical bone loss in the metacarpal diaphysis, but the decrease in cortical thickness and increase in total CSA in RA patients were even more pronounced. We attributed these findings to inflammation-enhanced ageing process [29]. In the present study, we found femoral cortical area, thickness and femoral index to decrease with time since menopause. Such age-associated changes in bone geometry have been attributed to estrogen deficiency-accelerated endocortical resorption with diminished periosteal apposition [30]. Periosteal apposition is considered as a compensatory mechanism to counterbalance buckling susceptibility, or as a coupling process between inflammation-induced endosteal osteoclastogenesis and periosteal osteoblastogenesis. Since both mechanisms are supposed to act at the same time, changes can be designated as an “inflammatory drift” [7, 29]. In both our previous studies, however, women treated with BP were excluded. Thus, the effects of BP on bone shaft geometry and vBMD in RA patients are not well described.

When investigating the effect of BP therapy on femoral bone, we found cortical CSA to be significantly decreased in patients with BP treatment while cortical thickness, medullary cavity and femoral bone CSA were similar between the groups. These observations leave the possibility that BP treatment may not fully inhibit inflammation-driven endosteal resorption in RA patients. Possible explanations for a lack of effect of BP on bone shaft geometry are the site specificity of BP, since the effect of BP is more likely to be seen in the trabecular bone [31], the difficulty to measure an effect of any therapy on bone shaft geometry [8], and the reduced effectiveness of BP in a low bone turnover state [32], particularly in patients on small GC dose and low disease activity, as in our study.

Our results are in agreement with those of Tournis et al. who evaluated tibial bone geometry in 65 postmenopausal osteoporotic RA patients treated with BP comparing it to that in women with primary osteoporosis [12]. At 38 % (cortical site), cortical CSA, and thickness were significantly lower in the RA group, whereas vBMD was comparable. Since periosteal circumference was comparable and endosteal circumference significantly higher, these results may be interpreted as ongoing endosteal trabecularisation and loss of cortical bone despite BP therapy. Concordant results were reported by Chen et al., who compared long-term BP users with therapy-naïve controls. They found BP users to have significantly thinner cortices than controls [33]. On the other hand, Zanchetta et al. compared the bone microarchitecture of distal radius and tibia in patients under BP treatment, treatment-naïve patients and patients with AFF and found no difference between the groups in cortical area or density [34]. Comparable bone resistance to bending and torsion calculated as polar SSI at 33 % of femoral diaphysis in our study may support the findings of Zanchetta et al. at radius and tibia.

There is still controversy on whether bisphosphonates change cortical bone geometry. In order to assess the effects of BP on bone geometry, we included muscle CSA around the proximal thigh, particularly since muscle force and loading have a high impact on bone adaptation [35]. When adjusted for determining factors, we found cortical thickness, cortical CSA and femoral index to highly correlate with muscle CSA.

In our study, muscle CSA at 33 % of femur was 13 % lower in patients under BP treatment compared to BP-naïve patients. These results are in agreement with recently published data by Uchiyama et al. who reported that BP therapy was associated with lower skeletal muscle mass at the proximal femur [36]. Chen et al. suggest that the impaired muscle mass in BP users may be due to comorbidities such as arthritis, asthma and osteoporosis, which have been reported to be associated with impaired muscle mass and strength [37]. Another factor is long-term GC therapy, the duration and dosage of which is most likely higher in BP users and causes wasting of proximal skeletal muscles [38]. In addition, alendronate has been shown *in vitro* to attenuate muscle regeneration capacity by preventing migration, proliferation and differentiation of undifferentiated human myogenic cells [39]. Since in our study, BP users had lower muscle CSA at 33 % of femur, we assume they are physically less active, generally in a poorer state of health and possibly have more comorbidities. Geometric differences of the femoral diaphysis that lead to AFF are therefore more likely to be caused by factors other than BP therapy and likely to be associated with altered unbalanced tensile strain pattern in the lateral femoral shaft during walking [40], an underlying hip geometry [41, 42], or due to a larger tibio-femoral angle [43], which then may place extra demands on the diaphyseal bone against resistance to bending and torsion.

## Strengths

The strength of this study is the method and site of measurement. Firstly, by measuring with pQCT, we were able to measure bone geometry such as total and cortical area, periosteal and endosteal circumference and cortical vBMD. Measurement of muscle area, a surrogate of muscle force, assesses differences in bone geometry in relationship to muscle force. Secondly, by choosing the site of femoral diaphysis, we were in close vicinity of the site where changes in bone density and geometry were measured in patients with of atypical femoral fractures.

## Limitations

The main limitation of our study is the small sample size. Since therapy was given only to patients with osteoporosis or increased fracture-risk, differences between the groups are likely to be confounded by this (potential) imbalance and it is difficult to distinguish the effect of the therapy from these confounding factors. Longitudinal studies are needed to further clarify the effect of BP on changes of muscle cross-sectional area, bone geometry, density and bone strength.

## Conclusion

The results of this study demonstrate that differences in bone femoral geometry between menopausal RA patients under long-term BP therapy and those not receiving BP are dependent on muscle area around the proximal thigh, age and time since menopause. No regression model showed BP therapy to have any significant impact on bone geometry, density or bone strength of the femoral diaphysis at 33 %.

## Abbreviations

AFF, atypical femoral fracture; BMD, bone mineral density; BP, bisphosphonate; CSA, cross-sectional area; GC, glucocorticoid; pQCT, peripheral quantitative computed tomography; RA, rheumatoid arthritis; SSI, stress–strain index; vBMD, volumetric bone mineral density
